# Successful Allergen-Specific Immunotherapy: Induction of Unresponsiveness by ‘Vaccination’

**DOI:** 10.3390/vaccines11121852

**Published:** 2023-12-14

**Authors:** Martin F. Bachmann, Monique Vogel, Daniel E. Speiser

**Affiliations:** 1 Department of Biomedical Research (DBMR), University of Bern, 3008 Bern, Switzerland; monique.vogel@unibe.ch (M.V.); daniel.speiser@unil.ch (D.E.S.); 2 Department of Rheumatology and Immunology, University Hospital of Bern, 3010 Bern, Switzerland; 3 Nuffield Department of Medicine, The Jenner Institute, University of Oxford, Oxford OX1 2JD, UK; 4 Department of Oncology, Lausanne University Hospital and University of Lausanne, 1066 Lausanne, Switzerland

## Abstract

The mechanisms of action of allergen-specific immunotherapy (AIT) are often referred to as the induction of ‘tolerance’. However, immunological ‘tolerance’ is defined as an alteration in the function or composition of immune cells. For AIT, this is not always the case, because it can also induce allergen-specific IgG antibodies that block allergic responses. To include all possible mechanisms that may mediate successful AIT, it is advantageous to use the scientific term ‘unresponsiveness’ instead of ‘tolerance’. In praxis, the term ‘vaccination’ is also appropriate, as AIT medications are specialized vaccines.

IgE-mediated type I hypersensitivity is often treated successfully with AIT, which comprises repetitive allergen injections over weeks to years and results in reduced allergic symptoms over time. The therapeutic benefit of AIT is typically long lasting and therefore disease modifying. In the early days of AIT, allergens were considered toxins and AIT was therefore considered a normal vaccination program, with the caveat that low doses of the allergen needed to be utilized to avoid ‘toxic’ side effects [1,2]. The discovery of IgE in 1968 by Ishizaka and Johansson fundamentally changed this perception [3], and the induction of ‘tolerance’ became the desired objective [4]. Within the framework of an aberrant IgE-mediated immune response, it was indeed reasonable to conclude that this immune response needed to be tolerized. However, for the reasons outlined below, we believe that the term ‘unresponsiveness’ is better suited to accurately describing the clinical efficacy of AIT than ‘tolerance’.

From an immunological point of view, the induction of tolerance reflects the modification of a cellular state by which lymphocytes are deleted from the system, become anergic/tolerized, or are suppressed by regulatory T cells [5,6,7] (Figure 1). Hence, tolerance reflects an active process in the immune system in order to avoid unwanted immune responses at the cellular level. The term ‘unresponsiveness’ does not invoke any type of mechanism regarding the unresponsiveness of the system, but simply measures and describes a lack of response [8]. In the case of AIT, the measured absence of a response is an allergic response. Thus, the term ‘unresponsiveness’ in allergy simply indicates an absence of response rather than implying a specific tolerizing mechanism.

A case in point is the B cell response to soluble proteins. Namely, if you try to immunize a host via the injection of a self-protein (e.g., cytokine), no antibody response will be mounted, which is because B cells generally do not respond when exposed to a pure self-protein [9,10]. In contrast, if a foreign T helper (Th) cell epitope is added to the same protein, a strong, high-affinity antibody response will be mounted against this protein [11,12,13]. Therefore, it is not the B cells that are tolerant; instead, T cell tolerance regulates the Th cell-dependent B cell responses for soluble antigens [14]. Thus, the observed absence of an antibody response after immunization with a pure protein is termed B cell ‘unresponsiveness’ and not ‘tolerance’, as the underlying mechanism may not necessarily be known.

The same is true of the reduced allergic responses observed after successful AIT. It is possible that relevant lymphocyte subsets have been deleted or rendered unresponsive or that regulatory T cells inhibit allergen-specific immune responses at the effector stage or in the long term; all of these represent active tolerizing mechanisms [15]. On the other hand, it has long been established that AIT induces allergen-specific IgG antibody responses that protect patients from allergic reactions. These antibodies either neutralize the allergen directly [16,17] or engage the inhibitory receptor FcγRIIb on effector cells [18,19]. IgG-mediated inhibitory mechanisms act at the level of the allergen and not lymphocyte downregulation; therefore, they do not fall under the umbrella of tolerance. In fact, the immune system is unresponsive due to allergen-specific IgG antibodies, which are responsible for the inhibition of allergic reactions through the above-mentioned mechanisms (via the direct neutralization of the allergen or by engaging the inhibitory receptor FcγRIIb).

Protective IgG antibodies are regularly found in patients treated with AIT and are the optimum correlate of successful therapy [20,21,22]. Therefore, this therapy is reminiscent of classical vaccination. The antigens are always specific ingredients in vaccines, as is the case for AIT allergens. In other words, vaccines against pathogens (e.g., Polio virus) contain antigens from the corresponding pathogen [23]. The term ‘vaccination’ is also suitable for AIT because it is familiar to everyone, and easier to understand in praxis than the scientific term ‘unresponsiveness’.

In summary, the exact mechanism of a given AIT is often not evident, and it may be possible that multiple mechanisms contribute to the success of AIT [6,15,24]. In addition to tolerance, allergic reactions may be inhibited by allergen-specific IgG antibodies [16,17,18,19]. Therefore, ‘unresponsiveness’ is scientifically more appropriate than ‘tolerance’ as an overarching term that describes the mechanisms of AIT. In praxis, the well-known term ‘vaccination’ may be used as it appropriately describes AIT.

## Figures and Tables

**Figure 1:**
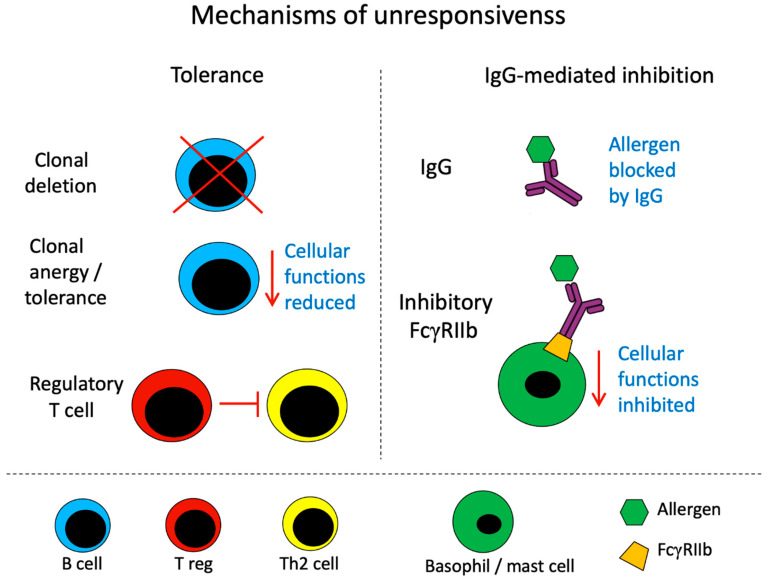
Mechanisms of unresponsiveness. Tolerance (**left**) occurs when the overall B cell function is reduced. This is achieved through the deletion of specific B cell clones, the permanent downregulation of specific B cell function (induction of anergy or tolerance), or via specific suppression through regulatory T cells (Treg). These mechanisms reduce allergen-induced immune responses. IgG-mediated inhibition (**right**) occurs through neutralization of the allergen by specific IgG, or through the binding of allergen-specific antibodies to FcγRIIb, which inhibits basophil/mast cell functions. Specifically, the ligation of IgG to allergens prevents them from binding to FcεRI-bound IgE, and the co-ligation of FcγRIIb and FcεRI by IgG-allergen immune complexes results in the inhibition of basophil/mast cell functions. The figure represents principal mechanisms that are simplified by omitting many additional facts. For more details, we refer to the literature as mentioned and cited in the text.
